# Supplementary figures and images for: Epidemiological consequences of enduring strain-specific immunity requiring repeated episodes of infection
Source: PLoS Comput Biol. 2020 Jun 5;16(6):e1007182. doi: 10.1371/journal.pcbi.1007182 (PMC7299408; doi:10.1371/journal.pcbi.1007182)

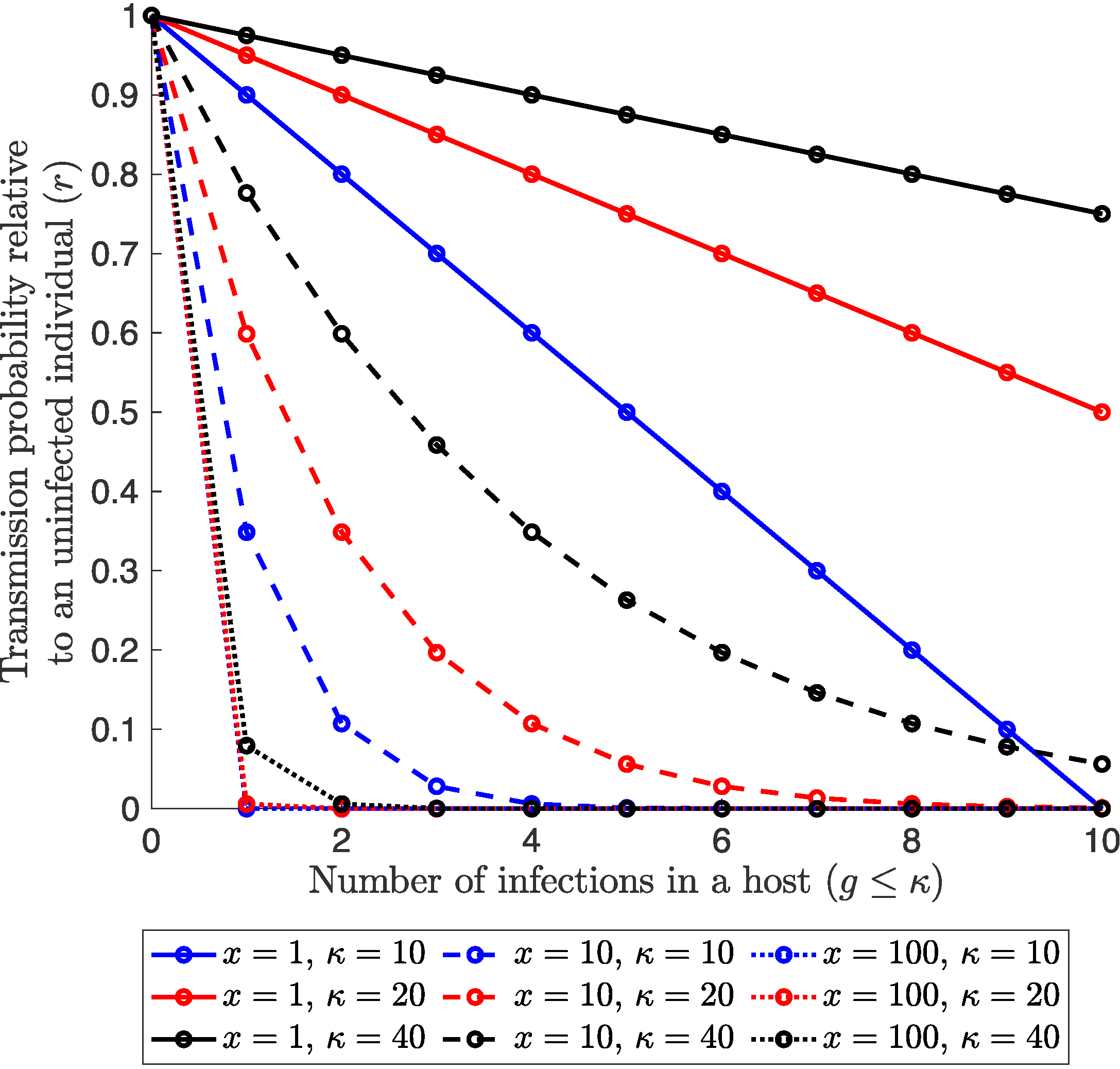

Supplement: S1 Fig — The relative probability r(g) of transmission to an infected host compared to an uninfected host is a function of the number of infections in the host g (horizontal axis). The co-infection carrying capacity κ and the level of resistance to co-infection x determine the shape of r(g). Here, r(g) is shown for 0 ≤ g ≤ 10, κ ∈ {10, 20, 40} and x ∈ {1, 10, 100}. (TIF) [file pcbi.1007182.s001.tif]

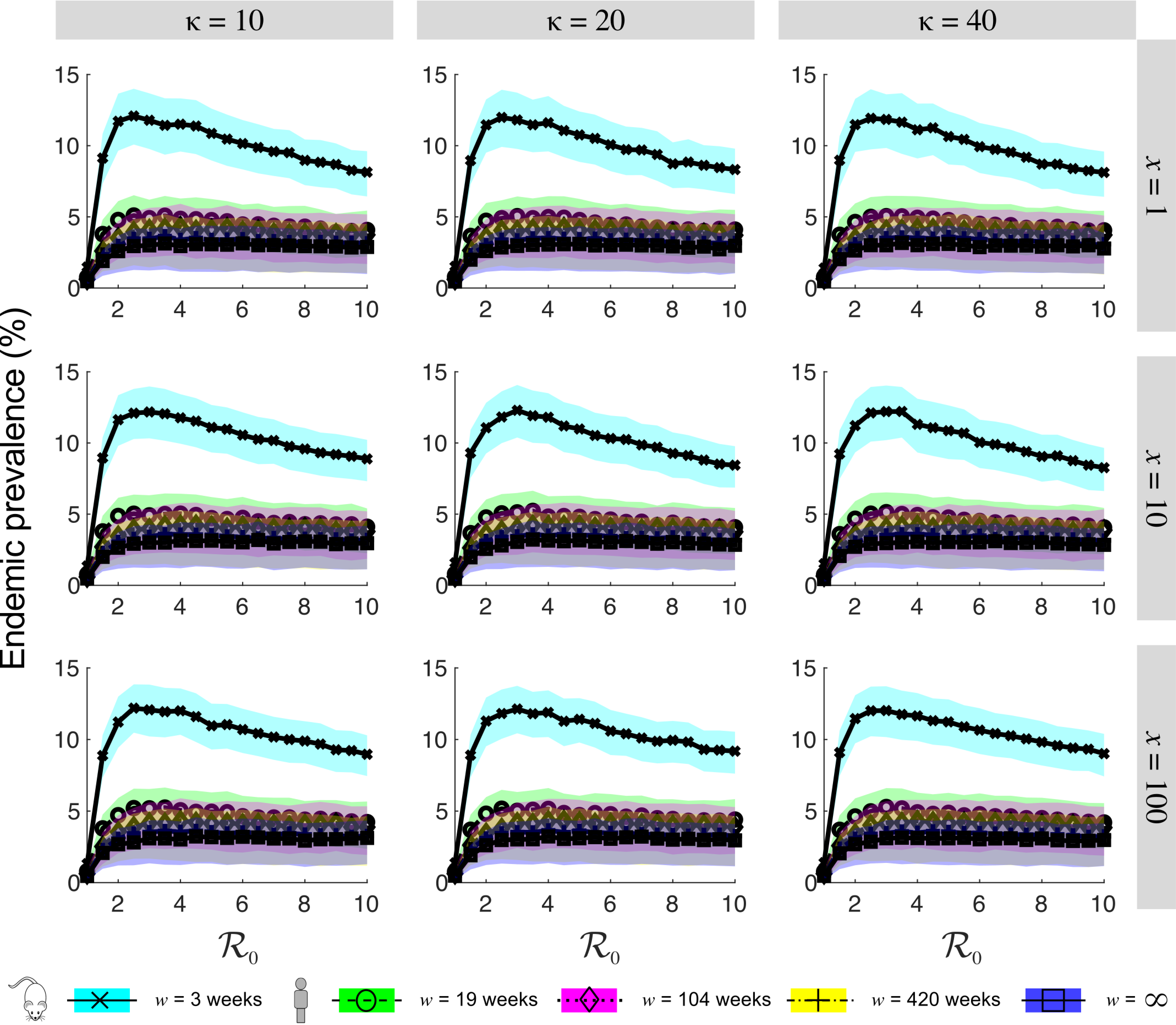

Supplement: S2 Fig — The mean (lines) and the interquartile ranges (shaded regions) of the total endemic prevalence of infected hosts P* from 80 simulations of the model, as a function of the basic reproduction number R0 (horizontal axis), for different values of the maximum inter-infection interval w (varied within each figure panel), the co-infection carrying capacity κ (varied across columns) and the level of resistance to co-infection x (varied across rows). Here, R0∈[1,10], 1/γ = 2 weeks, α = 0.002 per capita per week, c = 33, nmax = 40, n(0) = 30, N = 2500, σ = 0.9, ω1 = ω2 = 0.1, w ∈ {3, 19, 104, 420, ∞} weeks, κ ∈ {10, 20, 40} and x ∈ {1, 10, 100}. Note that the interquartile ranges overlap for w > 3 weeks. (TIF) [file pcbi.1007182.s002.tif]

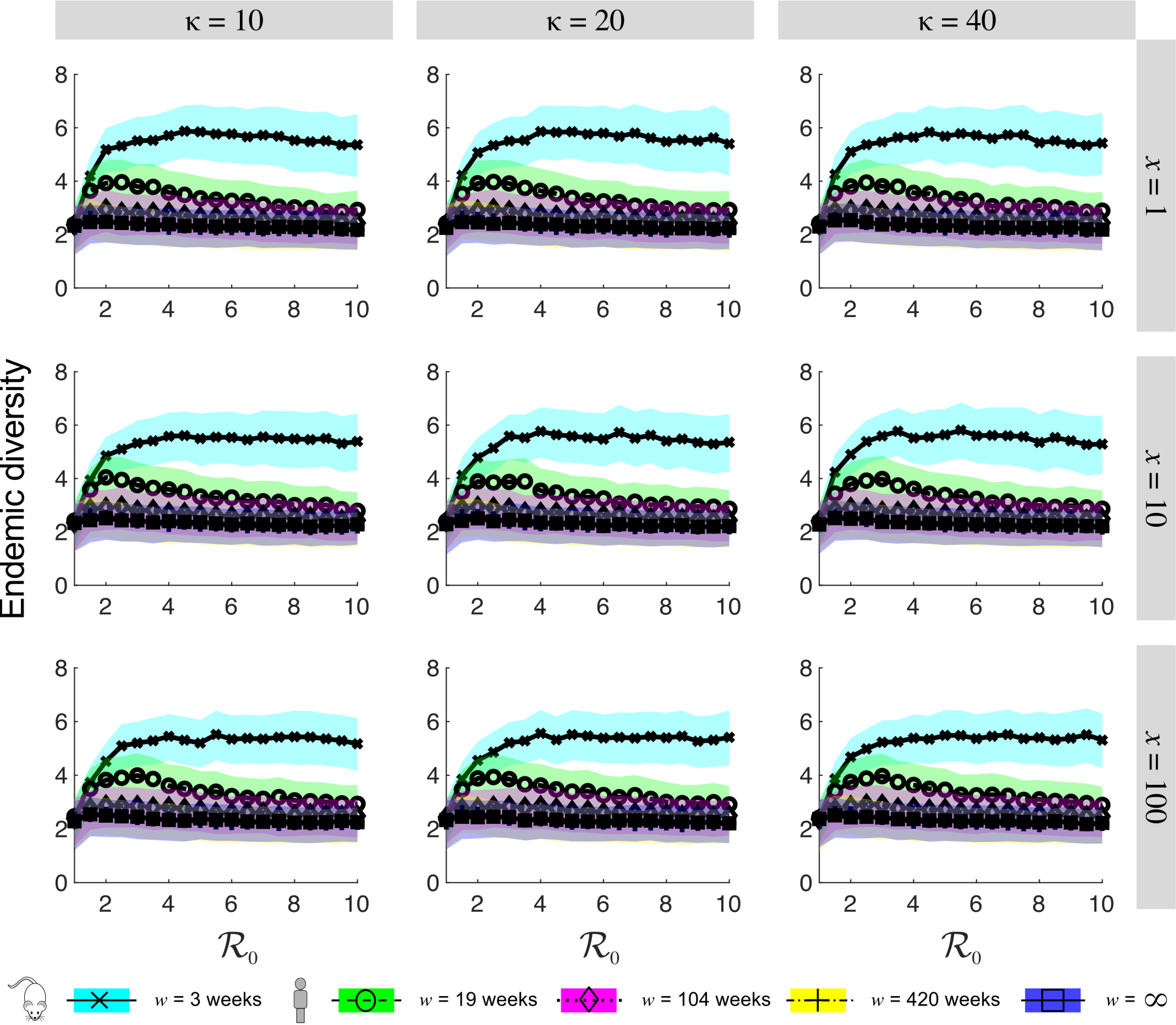

Supplement: S3 Fig — The mean (lines) and the interquartile ranges (shaded regions) of the endemic strain diversity D* from 80 simulations of the model, as a function of the basic reproduction number R0 (horizontal axis), for different values of the maximum inter-infection interval w (varied within each figure panel), the co-infection carrying capacity κ (varied across columns) and the level of resistance to co-infection x (varied across rows). Here, R0∈[1,10], 1/γ = 2 weeks, α = 0.002 per capita per week, c = 33, nmax = 40, n(0) = 30, N = 2500, σ = 0.9, ω1 = ω2 = 0.1, w ∈ {3, 19, 104, 420, ∞} weeks, κ ∈ {10, 20, 40} and x ∈ {1, 10, 100}. Note that the interquartile ranges overlap for w > 3 weeks. (TIF) [file pcbi.1007182.s003.tif]

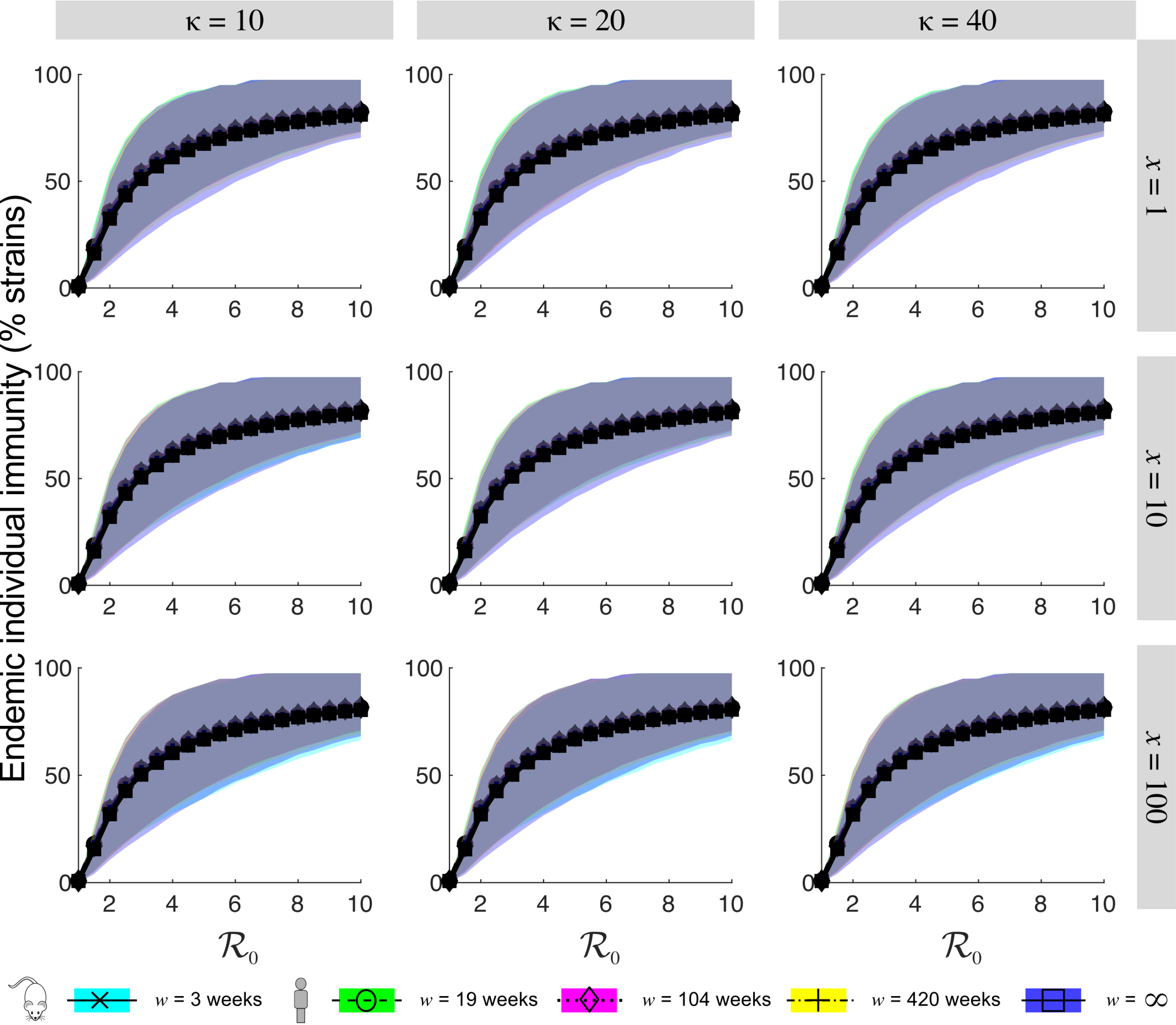

Supplement: S4 Fig — The mean (lines) and the interquartile ranges (shaded regions) of the mean endemic level of population immunity Y^* from 80 simulations of the model, as a function of the basic reproduction number R0 (horizontal axis), for different values of the maximum inter-infection interval w (varied within each figure panel), the co-infection carrying capacity κ (varied across columns) and the level of resistance to co-infection x (varied across rows). Here, R0∈[1,10], 1/γ = 2 weeks, α = 0.002 per capita per week, c = 33, nmax = 40, n(0) = 30, N = 2500, σ = 0.9, ω1 = ω2 = 0.1, w ∈ {3, 19, 104, 420, ∞} weeks, κ ∈ {10, 20, 40} and x ∈ {1, 10, 100}. Note that all interquartile ranges overlap. (TIF) [file pcbi.1007182.s004.tif]

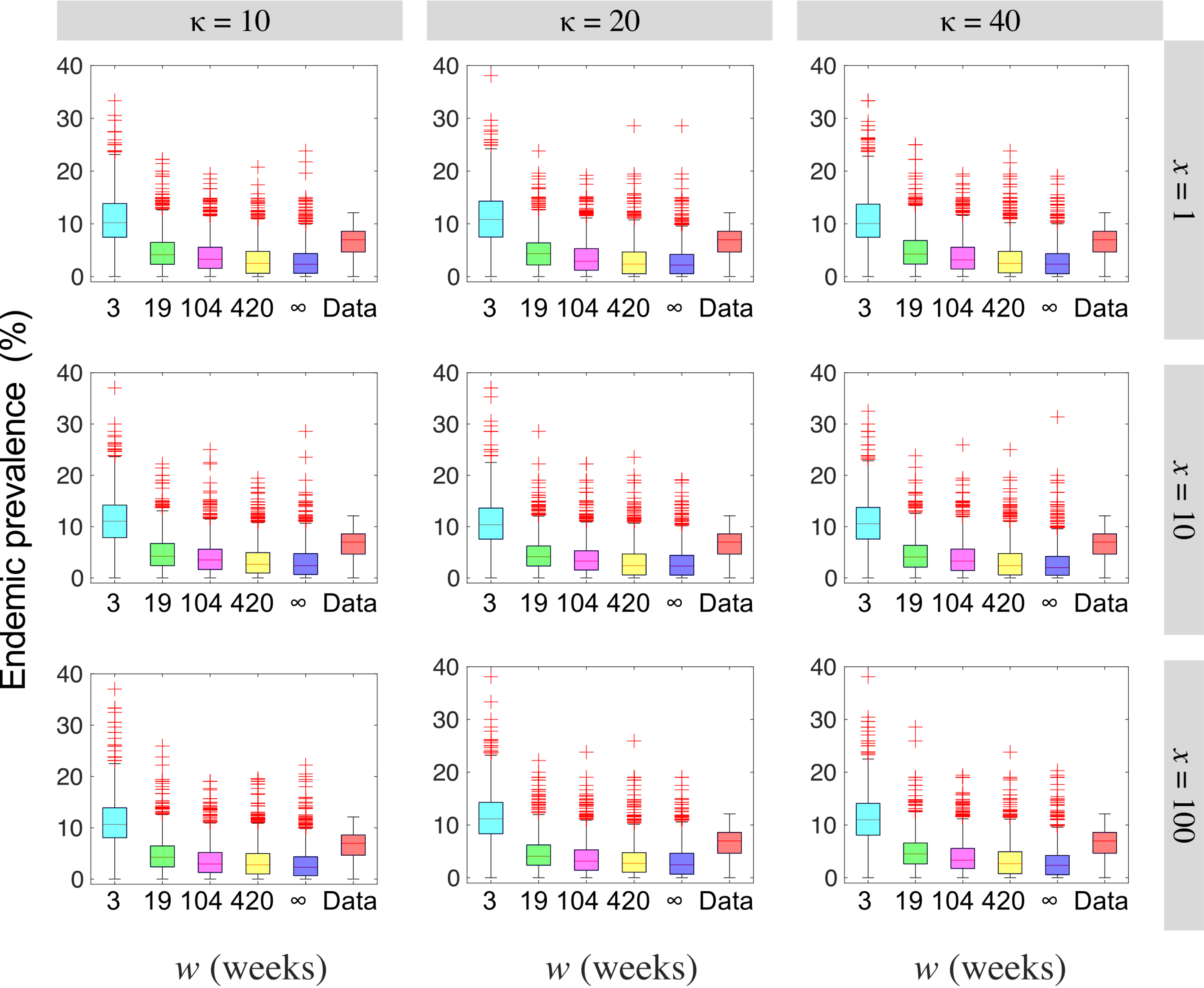

Supplement: S5 Fig — Model outputs are generated for a range of values of co-infection parameters κ (varied across columns) and x (varied across rows), and the inter-infection infection interval w (varied within each figure panel). Distributions of P* for each parameter combination were obtained from 80 simulations of the model. Here, R0=2.5, 1/γ = 2 weeks, α = 0.002 per capita per week, c = 33, nmax = 40, n(0) = 30, N = 2500, σ = 0.9, ω1 = ω2 = 0.1, κ ∈ {10, 20, 40}, x ∈ {1, 10, 100}, and w ∈ {3, 19, 104, 420, ∞} weeks. (TIF) [file pcbi.1007182.s005.tif]

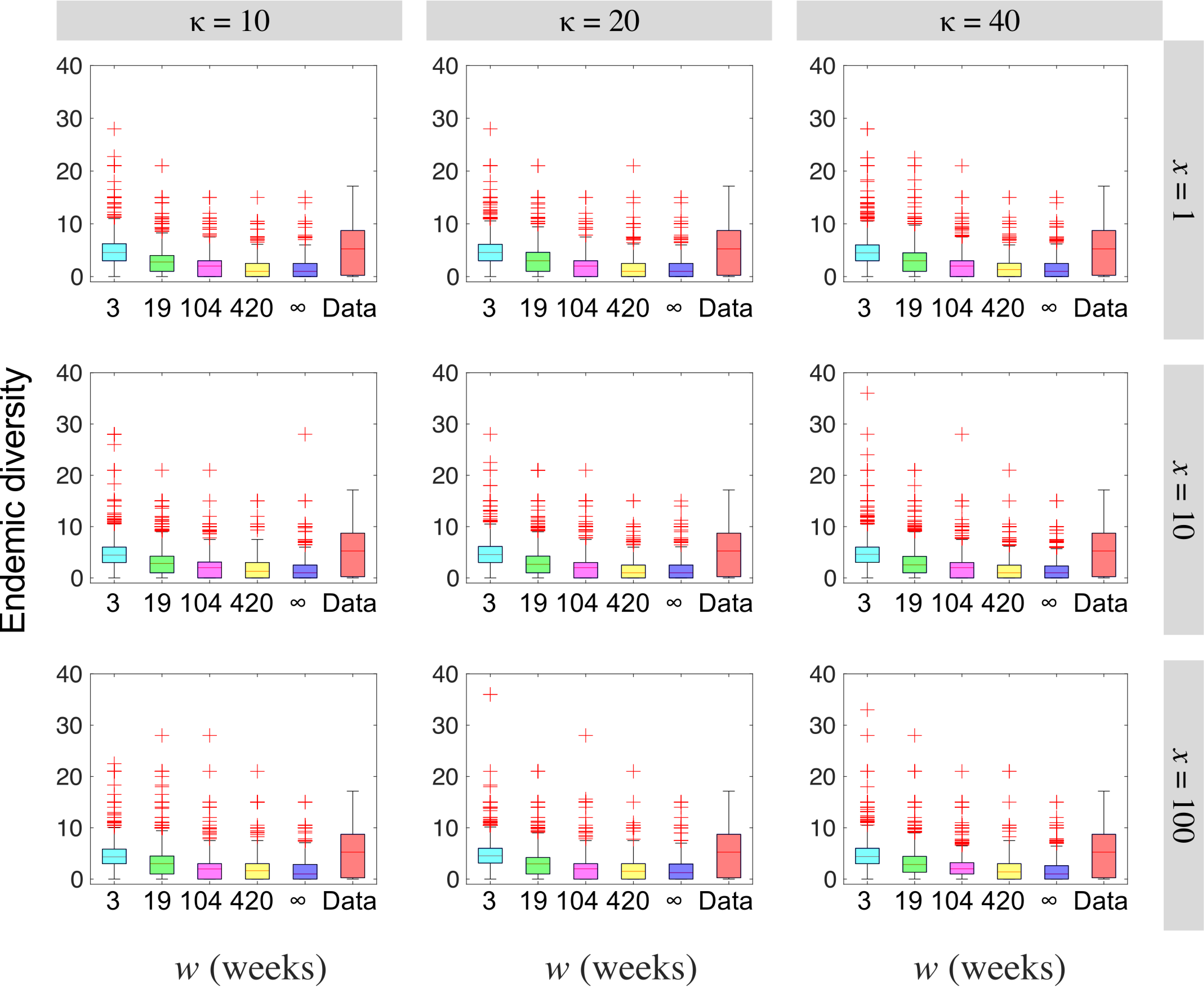

Supplement: S6 Fig — Model outputs are generated for a range of values of co-infection parameters κ (varied across columns) and x (varied across rows), and the inter-infection infection interval w (varied within each figure panel). Distributions of D* for each parameter combination were obtained from 80 simulations of the model. Here, R0=2.5, 1/γ = 2 weeks, α = 0.002 per capita per week, c = 33, nmax = 40, n(0) = 30, N = 2500, σ = 0.9, ω1 = ω2 = 0.1, κ ∈ {10, 20, 40}, x ∈ {1, 10, 100}, and w ∈ {3, 19, 104, 420, ∞} weeks. (TIF) [file pcbi.1007182.s006.tif]
